# Moisture Absorption Effects on the Mechanical Properties of Sandwich Biocomposites with Cork Core and Flax/PLA Face Sheets

**DOI:** 10.3390/molecules26237295

**Published:** 2021-12-01

**Authors:** Hom Nath Dhakal, Chulin Jiang, Moumita Sit, Zhongyi Zhang, Moussa Khalfallah, Erwan Grossmann

**Affiliations:** 1 Advanced Polymers and Composites (APC) Research Group, School of Mechanical and Design Engineering, University of Portsmouth, Portsmouth PO1 3DJ, UK; chulin.jiang@port.ac.uk (C.J.); moumita.sit@port.ac.uk (M.S.); zhongyi.zhang@port.ac.uk (Z.Z.); 2 Kairos, 1 Rue des Senneurs, ZI du Moros, 29900 Concarneau, France; moussa@kairos-jourdain.com (M.K.); biocomposite@kairos-jourdain.com (E.G.)

**Keywords:** biocomposites, flax fibres, mechanical properties, impact damage, delamination

## Abstract

The aim of this study was to evaluate the moisture absorption behaviour and its influence on the mechanical properties of newly developed sandwich biocomposites with flax fibre-reinforced poly-lactic acid (PLA) face sheets and soft cork as the core material. Three different types of sandwich biocomposite laminates comprised of different layup configurations, namely, non-woven flax/PLA (Sample A), non-woven flax/PLA and cork as core (Sample B) and non-woven flax/paper backing/PLA, cork as core (Sample C), were fabricated. In order to evaluate the influence of moisture ingress on the mechanical properties, the biocomposites were immersed in seawater for a period of 1200 h. The biocomposites (both dry and water immersed) were then subjected to tensile, flexural and low-velocity falling weight impact tests. It was observed from the experimental results that the moisture uptake significantly influenced the mechanical properties of the biocomposites. The presence of the cork and paper in sample C made it more susceptible to water absorption, reaching a value of 34.33%. The presence of cork in the core also has a considerable effect on the mechanical, as well as energy dissipation, behaviours. The results of sample A exhibited improved mechanical performance in both dry and wet conditions compared to samples B and C. Sample A exhibits 32.6% more tensile strength and 81.4% more flexural strength in dry conditions than that in sample C. The scanning electron microscopy (SEM) and X-ray micro-CT images revealed that the failure modes observed are a combination of matrix cracking, core crushing and face core debonding. The results from this study suggest that flax/PLA sandwich biocomposites can be used in various lightweight applications with improved environmental benefits.

## 1. Introduction

The advancement of high-performance carbon and glass fibre-reinforced polymer composites has changed our society in many ways. These materials have excellent performance and have been immensely successful over the last few decades in terms of providing lightweight composite materials in various critical applications in comparison to their metallic counterparts. However, these reinforcements are fossil fuel based; their adverse effects, such as resource depletion, global warming and an overall negative impact on the environment, are major concerns. In recent years, there has been increased awareness all over the world regarding the negative influence of such materials on global warming and the environment. Over the last decade, composites reinforced with natural fibres have received ever increasing attention, both from the academic world and from the various industries [1,2]. Extensive research works have been conducted in the past, focusing on the development and characterisation of composites using natural fibres as reinforcements in thermoset and thermoplastic polymer matrices [3,4,5,6]. Natural fibre-reinforced biocomposites are becoming more popular due to their environmentally friendly, acceptable specific properties, comparatively lower cost and biodegradable attributes. Additionally, the environmental performance indicated by the life cycle assessment (LCA) shows that natural fibres reinforced biocomposites are superior compared to that of conventional composites [7,8]. However, to attain fully biodegradable composites, both the matrix and the reinforcements are expected to be from renewable sources. Natural fibres have shown their potential and can be a viable substitute to widely used glass and carbon fibres in composite reinforcements as these are natural, renewable and abundant [9,10,11]. The work carried out by Yuanjian and Isaac on the mechanical properties of non-woven hemp fibre mat reinforced polyester composites reported that the tensile properties of hemp polyester were comparable to those of glass fibre composites. The tensile strength and modulus of 44 wt.% of fibre were 53 MPa and 6.2 GPa, respectively. For glass fibre-reinforced composites with 42 fibre wt.%, the tensile strength and modulus were recorded at 43 MPa and 5.9 GPa, respectively [12].

Due to the high stiffness/strength-to-weight ratios, improved bending stiffness and lightweight, sandwich composites, the structures have been applied in many engineering applications, such as marine, building materials, transportation and aircraft structures [13]. The face sheets of sandwich composites used in various applications are normally constructed using glass or carbon fibre due to their high mechanical properties. Due to this factor, among others, biobased sandwich composites are used in low load-bearing interior floor applications in automotive and marine sectors. In order to make composites more sustainable, researchers are increasingly investigating using renewable, biodegradable materials to replace the conventional glass/carbon fibre-petrochemical based resin composites. Therefore, “green” sandwich composite structures have attracted many interests [14,15,16,17]. Among these green composites, the flax-reinforced biobased composites have been widely studied in recent years [18,19,20,21] due to the fact that flax fibre possesses high strength and stiffness in comparison to other natural plant fibres. For the “skin” material of the sandwich structure, flax fibres can be attractive alternatives due to the excellent properties of flax fibres [22]. For the “core” material, cork, a cellular material obtained from the bark of Cork Oak trees, can serve as an ideal material because it is 100% biodegradable and renewable with advantages, including low density, high shear modulus, damping ratio and impact resistance. Moreover, from the environmental point of view, flax fibres possess significantly lower embodied energy in comparison to glass and carbon fibres [23]. 

Additionally, compared with glass and carbon fibre-reinforced composites, the superior damping properties of flax fibre composites have been reported in many studies [24,25,26,27,28,29,30,31]. The improved damping properties could be attributed to the complex microstructure of flax fibres, which promotes energy dissipation through firstly; friction between cellulose, lignin and hemicellulose in each cell wall and secondly; friction between the cell walls [25,27,31]. The natural fibres possess relatively high specific mechanical properties due to their low densities and very high performances of dynamic behaviour [32]. A comparative study carried out by Huda et al. reported that the tensile strength and modulus of glass/PLA was 80.2 MPA and 6.7 GPa, respectively, with 30 wt.% of glass fibre content. Similarly, their work highlighted that the tensile strength and modulus of flax/PLA (30 wt.% of flax fibre content) was found to be 53 MPa and 8.3 GPa, respectively. It can be seen from these results that flax/PLA showed higher modulus in comparison to glass/PLA composites [33].

There are not many comprehensive analyses reported on the mechanical properties of flax fibre-reinforced sandwich biocomposites apart from some limited work reported by Sarasini et al. and Mancuso, et al. [16, 22]. A sandwich composite is particularly useful in applications such as floor and wall panels, doors and other structural elements where superior flexural bending strength is required. A good impact toughness is also important in these applications, especially when used in automotive components. Despite many attractive attributes highlighted above, natural fibre-reinforced composites are highly susceptible to moisture absorption due to the strong hydroxyl groups present. Water ingress into the composite weakens the fibre matrix interface and interphase regions through various mechanisms affecting the overall performance and the durability of composites. In such cases, retaining its durability becomes crucial for these composites, especially if they are to be used in marine and transport applications. Furthermore, low-velocity impact damage, especially barely visible impact damage (BVID), arising from foreign objects impacts during their service life, causes delamination and reduction of the structural integrity of the composites. 

In the light of the above issues, this study aimed to design and develop fully green composite with sandwich structures, meeting some of the long-term durability requirements in marine applications, as well as in point-of-purchase (POP) sectors, due to their lightweight and high bending stiffness. To achieve this goal, biocomposites composed of non-woven flax fibre-reinforced polylactic acid (PLA) composites (Sample A), non-woven flax/PLA face sheets with cork core sandwich structure (Sample B), and non-woven flax face sheets/PLA with paper backing with cork core (Sample C) were constructed as sustainable fully green composite solutions. Furthermore, the influence of moisture absorption on the mechanical properties (tensile, flexural and low-velocity falling weight impact) and damage behaviour of flax/PLA face sheets and cork core with a paper layer sandwich panels are investigated by using scanning electron microscopy (SEM) and X-ray micro-CT. 

## 2. Experimental Details

### 2.1. Materials

The newly developed composite material (produced and provided by Kairos, France) is a sandwich structure with flax composite as the skins and cork as the core. Three composite layups (Sample A, sample B and sample C) with different configurations are used for the fabrication of different laminates. Sample A has the following layup: film PLA /non-woven flax/PLA/film PLA. This sample has 7 layers of non-woven flax (40 wt.%) with an areal density of 350 g/m^2^ without any paper and cork with 60 wt.% of PLA. A layer of PLA film of 350 µm is added to the outer surfaces. 

Sample B is a symmetric sandwich panel with cork as the core with the following layup:

(Film PLA/non-woven flax/PLA/cork)_s_ the material was prepared with a PLA film of 350 µm and non-woven flax of 350 g/m² based on 40 wt.% of flax and 60 wt.% of PLA. 

Sample C is also a symmetric sandwich panel with the following layup:

(Film PLA/non-woven flax/PLA/paper/non-woven flax/PLA/cork)_s_ the material was prepared with a PLA film of 350 µm and non-woven of 350 g/m² based on 40 wt.% of flax and 60 wt.% of PLA and a Kraft paper of 100 g/m². The fibre volume fraction (FVF) for sample A was 30%. For samples B and C, the FVF was 23%.

### 2.2. Fabrication of Biocomposite Laminates

**Figure 1:**
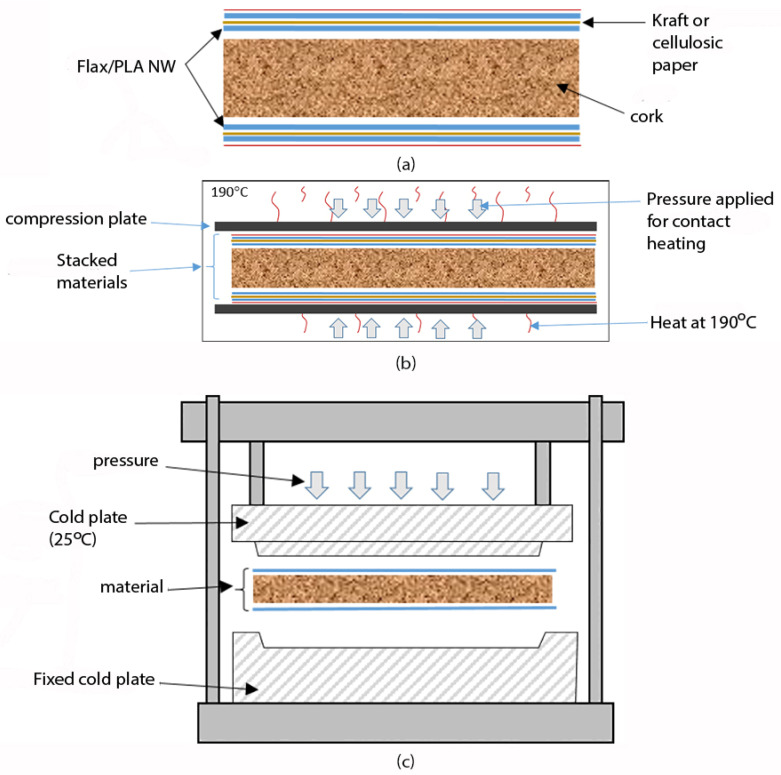
Fabrication process of the sandwich laminate. (**a**) Staking sequence of the biocomposites laminates; (**b**) Contact heating of the biocomposite laminates; (**c**) Compression and speed cooling system used of the consolidation of materials after heating.

The non-woven flax/PLA specimen was prepared by the compression moulding technique. The different steps involved to produce samples B and C of the sandwich laminate fabrication process are depicted in Figure 1.

Moreover, the fabrication process of different samples is presented in the following steps:

Step 1: Staking of the layers with respect to the following symmetric staking sequence (PLA film 350 µm/2 layers of flax + PLA NW (350 g/m²)/cork (with a density of 0.24 g/cm^3^)) 

Step 2: Contact heating of the staked layers by using a thermal compression machine at 190 °C for 5 min, under a pressure of 1 bar, with the respect of 3 degassing cycles. 

Step 3: The heated materials were then transferred to a compression and cooling machine for the consolidation of the different layers together, at 25 °C, for 10 min under a pressure of 3 bar. 

Similarly, the different samples for various characterisation (tensile testing, flexural testing, falling weight impact testing in both dry and wet conditions) were prepared from the obtained laminates.

### 2.3. Water Absorption Test

The composite samples were immersed in the seawater bathes for a time period until saturation was reached. Five specimens from each type of material are cut from the composite panels. A desiccator was used to dry all the samples at room temperature for 24 h before moving to the baths. In order for the samples to be fully submerged into the water, the cork was protected using silicone sealant around the edges to prevent the water from affecting the cork core directly. After every 24 h, the samples were removed from the seawater and weighed using a digital scale. This process was repeated over 30 days until the constant weight (saturation moisture uptake) of all the samples were obtained. 

For each composite type, five samples were immersed in the seawater, and their average was recorded. The percentage of moisture uptake was calculated by the weight difference between the samples immersed in water and the samples after desiccation using Equation (1).

(1) $$∆M\left(t\right)\left(\%\right)=\frac{m_{t}-m_{0}}{m_{0}}\times 100$$

where, ∆M(t)(%) is the percentage of moisture uptake at different time intervals, $m_{t}$ is mass of specimens during ageing (grams) and $m_{o}$ is mass of specimens before ageing (grams).

### 2.4. Mechanical Testing and Characterisation

Tensile, flexural and drop weight impact tests were employed to evaluate the influence of various layup configurations on the mechanical characteristics of the composites.

#### 2.4.1. Tensile and Flexural Testing

Tensile and flexural testing were performed on a Zwick/Roell Z030 universal machine using pull-to-break mode and bend-to-rupture modes, respectively. Tensile tests were performed at 2 mm/min; the tensile modulus, strength and strain to failure (STF) were provided by stress–strain curves. For the flexural testing, the test span and indenter motion rate were set at 50 mm (span-to-depth ratio of ~16) and the crosshead speed to 2 mm/min. The tests were conducted in accordance with the BS EN 2747 standard [34] for tensile characteristics and the BS 2782-10: Method 1005 standard [35] for flexural characteristics. The specimens were cut to the dimensions given by the standards. In order to investigate the moisture influence, at least 5 specimens from each laminate at dry and wet conditions were tested and an average was taken. All tests were conducted at room temperature (~23 °C).

#### 2.4.2. Low-Velocity Drop Weight Impact Testing

**Figure 2:**
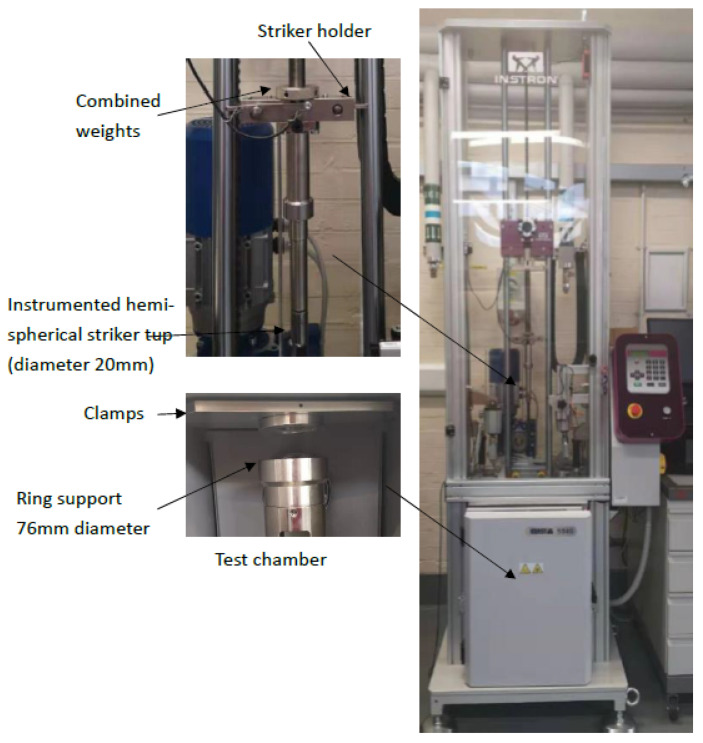
Impact testing system (Instron CEAST 9340 drop tower).

Low-velocity drop weight impact testing was conducted by using an Instron CEAST 9350 drop tower impact system, as depicted in Figure 2. The testing was conducted in accordance with the BS EN ISO 6603-2 recommendations [36]. A low-velocity falling weight impact was employed with a hemispherical nose impact tip with a diameter of 20 mm on the drop weight tower fitted in the impactor of a total mass of 3.15 kg. The test specimens were 100 × 100 mm squares. Five specimens were tested for each composite category comprised of dry and wet samples. The incident energy employed was 10 Joules, enough to perforate the specimens. The impacted samples were visually inspected to measure the visible damage. 

#### 2.4.3. Fractured Surface Characterisation by SEM

In order to investigate the damage mechanisms of the impacted samples, the images of the fractured surfaces of the impacted sandwich composites were acquired using a ZEISS EVO MA10 scanning electron microscope (SEM). The specimens were surface prepared with gold coating by using Quorum Q150R S coater to make them electrically conductive.

#### 2.4.4. X-ray Micro-CT

X-ray-computed micro-tomography (micro-CT) evaluations of the impacted samples were conducted using a Nikon (Xtec) XTH225 by reconstructing the three-dimensional (3D) images of the samples from a large number of X-ray projection images. 

## 3. Results and Discussion

### 3.1. Moisture Absorption Behaviour

**Figure 3:**
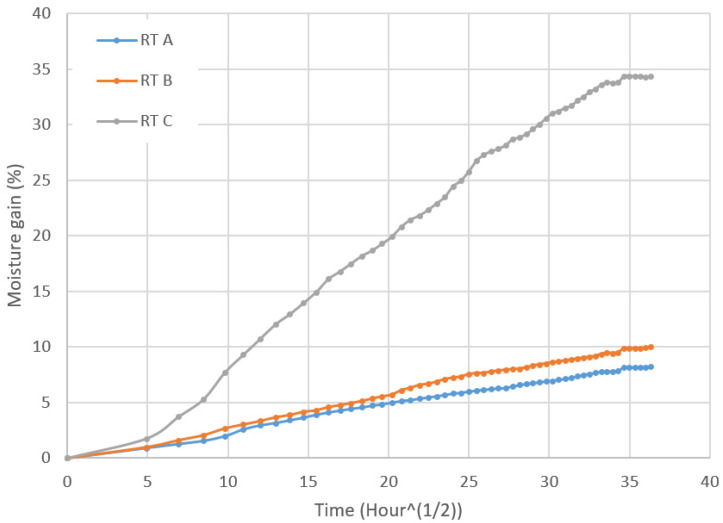
Water absorption curves for different biocomposites at room temperature.

Figure 3 depicts the percentage of the weight gain vs. a function of the square root of time for the three different biocomposites immersed in seawater at room temperature before the mechanical testing.

It can be seen from the moisture gain versus time traces that the moisture uptake at the beginning is linear and then rapidly increases for sample C compared to samples A and B. The moisture uptake reaches saturation for all the samples at 1200 h as the water absorption curves become a straight line, as depicted in Figure 3. The maximum weight gain percentages for samples A, B and C are approximately 8.22%, 10.04% and 34.33%, respectively. The noteworthy higher moisture uptake percentage for sample C is attributed to the porous effect of the paper and cork present in sample C. The reasons for the differences for moisture absorption for these three types of specimens are related to damage caused in the interface between the face sheet and the core. The edge of the specimens was sealed with sealant. However, the sealant layer may not have been sufficient to prevent the moisture ingress. Another factor contributing to high moisture could be related to surface defects. The moisture ingress from the face sheet can penetrate to the core creating a water travel pathway as a result of degradation of the flax-composite surface due to moisture absorption.

### 3.2. Tensile Properties

**Figure 4:**
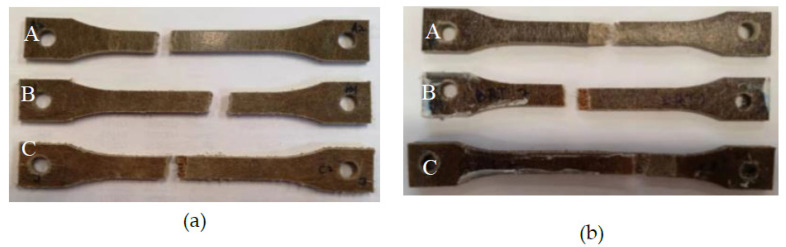
Typical fractured samples of three different biocomposite samples following tensile testing with (**a**) dry and (**b**) seawater immersed at room temperature.

**Figure 5:**
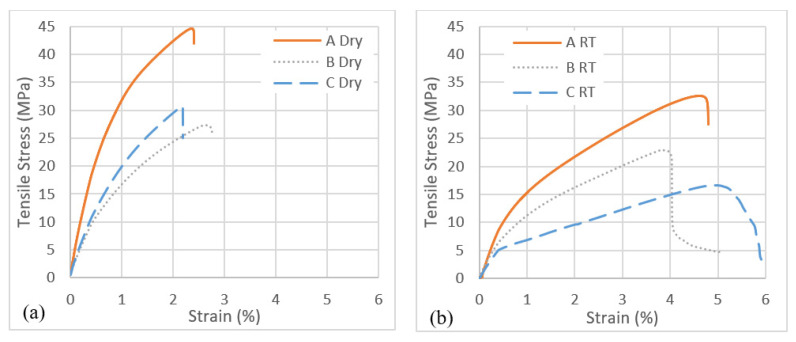
Tensile stress–strain curves from different biocomposites with different environmental conditions, (**a**) dry conditions and (**b**) seawater at room temperature.

The representative samples following the tensile test are presented in Figure 4. The stress–strain curves of the composite samples A, B and C are plotted graphically for different environmental conditions and depicted in Figure 5. The tensile modulus and strength of the composites are summarised in Table 1. It can be seen that sample C has the lowest tensile strength and modulus. This could be attributed to the presence of a layer of paper between the non-wovens, which can block the PLA impregnation in the cavities of the cork, thus reducing the strength and stiffness of the material. It is also evident from Table 1 that the tensile strength of the samples significantly degrades with seawater immersion. Generally, when natural fibres absorb moisture, they swell, which promotes adverse effects on the tensile properties due to the weak fibre matrix interface (Dhakal et al., 2007).

A comprehensive study was undertaken by Pantaloni et al. [37] on non-woven flax fibre-reinforced PLA, PHA (Polyhydroxyalkanoates), PP (Polypropylene) and PBS (Polybutylene succinate) biocomposites. Their study first reported that PLA, along with PHA and PBS, showed higher tensile strength than that of PP, as was taken as the industry reference material. Moreover, their results highlighted that flax/PLA exhibited the highest tensile modulus compared to other biocomposites, such as flax/PHA, flax/PBS and flax/PP. The key factors contributing to the highest tensile properties of flax/PLA is attributed to the quality of preform and the optimised manufacturing process used. They reported that the porosity was lower than 2%. These results confirmed that flax/PLA biocomposites are credible replacements to flax/PP. Additionally, it was argued that fibre morphology and preform architecture together with enhanced fibre matrix interface in order to transfer the stress from matrix to reinforcement was crucial for achieving the enhanced performance [37].

According to the tensile properties displayed in Table 1 and Figure 5, the tensile modulus and strength for composite A were significantly higher than that of samples B and C. Upon immersion in seawater, the tensile modulus and strength decreases by approximately 42% and 28%, respectively, in the case of sample A. Contrary to this, for sample B with moisture exposure, the tensile modulus and strength was decreased by approximately 11% and 19%, respectively. This phenomenon could be attributed to the presence of cork as the core material in sample B. The presence of cork played a significant role in preventing the fibre-matrix delamination, thus resulting in less reduction of the tensile properties. However, for sample C, this was not the case. With exposure to moisture, the modulus and strength were decreased by approximately 52% and 41%, respectively. The decrease in tensile properties for sample C after the moisture exposure is the highest compared to samples A and B. This behaviour could be attributed to higher moisture absorption by sample C, creating a weak fibre matrix interface as a result, reducing the tensile properties. The paper layer present in sample C is sensitive to moisture absorption, which, in turn, affects its properties and initiates delamination when subjected to tensile tests [38].

**Figure 6:**
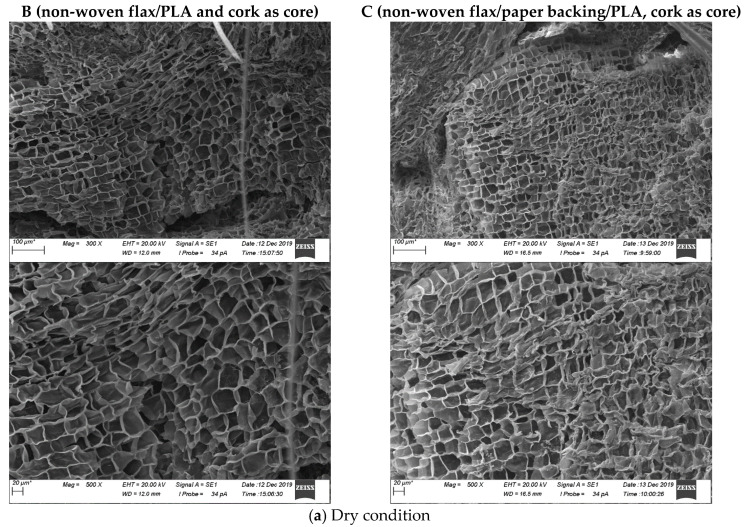

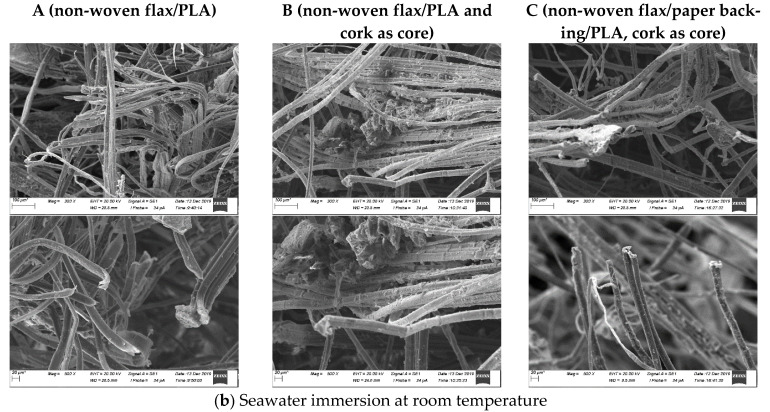
SEM micrographs of typical fracture sections of the sandwich composites after tensile testing at two levels of magnification; (**a**) the view of cork and (**b**) the view of the fibres in the skin material. **A** (non-woven flax/PLA); **B** (non-woven flax/PLA and cork as core); **C** (non-woven flax/paper backing/PLA, cork as core).

SEM images of fractured surfaces of material A, B and C are illustrated for different environmental conditions, such as dry and seawater immersion at room temperature in Figure 6. Fracture mechanisms, including matrix cracking, fibre fracture, fibre pull-out and cork cell breakage, can be observed from these images.

### 3.3. Flexural Properties

**Figure 7:**
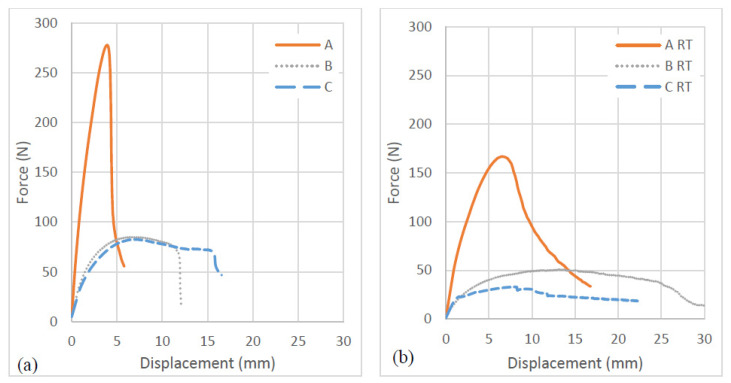
Force–displacement curves obtained from three-point bending test for samples A, B and C with different environmental conditions, (**a**) dry and (**b**) seawater immersed at room temperature.

The flexural behaviour of the composite samples is represented in the form of force–displacement curves obtained from the three-point bending tests. Typical load versus deformation traces is depicted in Figure 7. 

The average flexural strength and modulus of the composite samples for different environmental conditions are calculated employing standard formula using load versus deformation curves and are presented in Table 2. 

It can be observed (Table 2) that dry sample A specimens (without immersion in water) has a flexural modulus of 17.74 GPa and a flexural strength of 162.79 MPa. Sample B specimens showed a flexural strength of 48.30 MPa and modulus of 5.46 GPa. In the case of sample C, the average flexural strength and modulus were recorded as 30.21 MPa and 2.34 GPa, respectively. With exposure to moisture, however, the strength and modulus were decreased significantly. For sample A, for example, in the wet condition, the strength was recorded as 64.17 MPa (a decrease of 60.58%) and the modulus as 2.30 GPa, (a decrease of 87.03%). It can be observed that the flexural strength and modulus significantly decrease with moisture ingress. 

**Figure 8:**
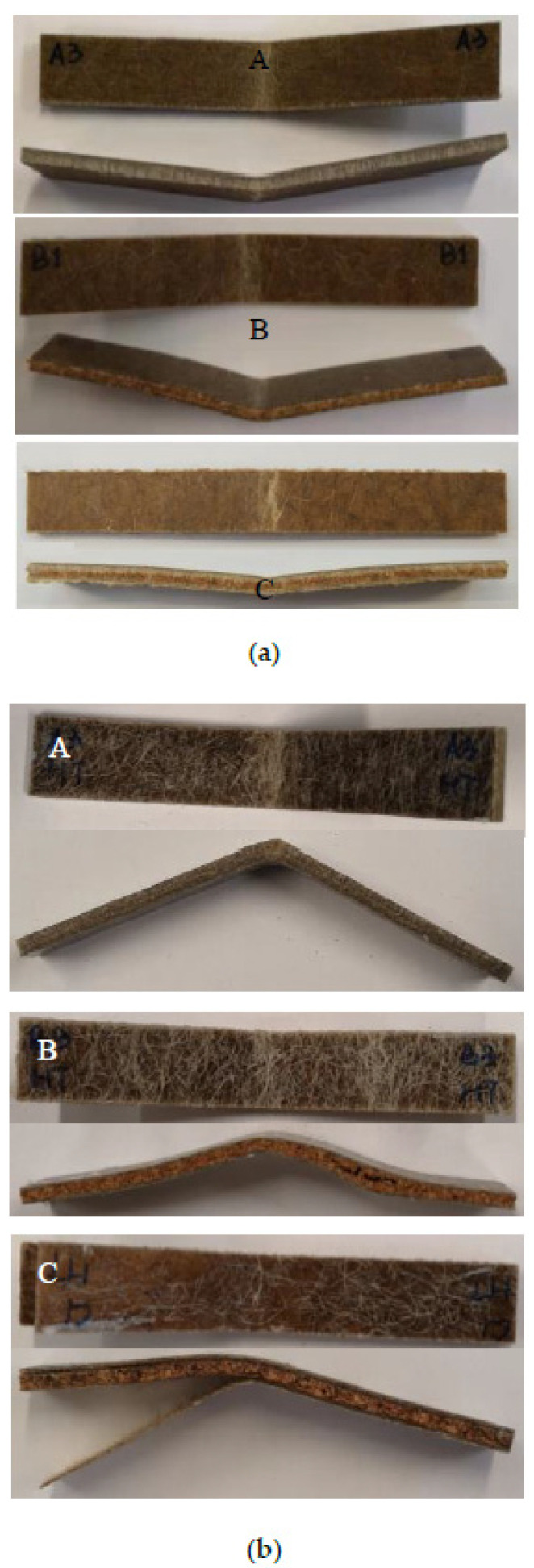
Damaged samples following flexural testing for different materials (**a**) dry and (**b**) seawater immersed at room temperature.

Figure 8 shows the representative samples following the three-point bending test. It is clear that the sample does not fracture during the bending testing; instead, a permanent plastic deformation is formed. The observation suggests ductile flexural characteristics of flax face sheet and cork core biobased sandwich composites. Contrary to tensile test results, in sample B, the decrease in flexural modulus and strength showed a higher order of magnitude. The weakening of face sheet-core adhesion due to moisture ingress is attributed to a significant loss of flexural properties for sample C compared to samples A and B.

### 3.4. Low-Velocity Drop Weight Impact Test Results

**Figure 9:**
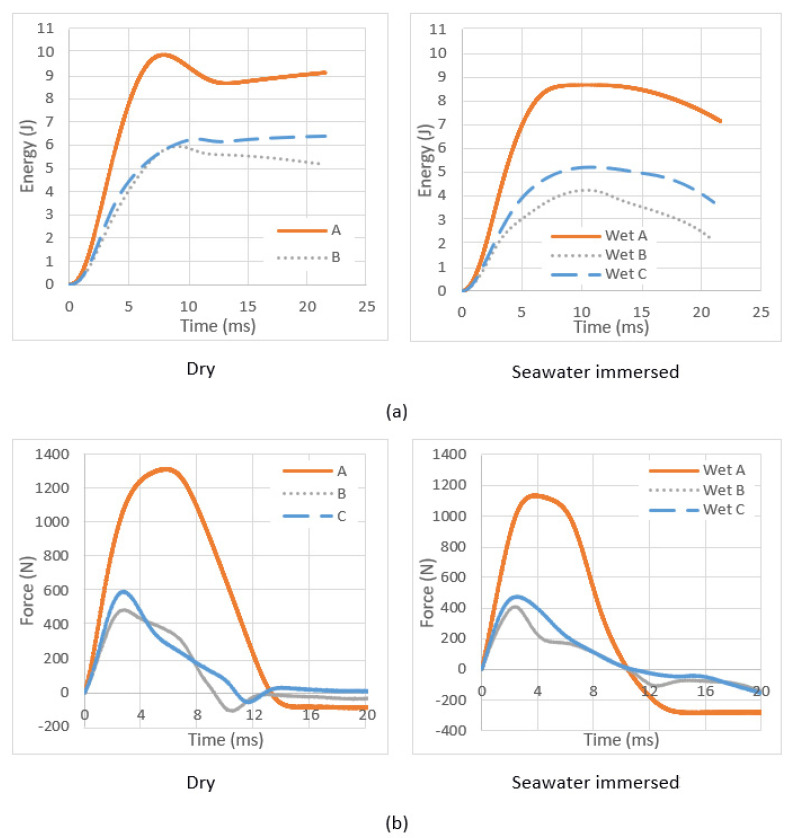

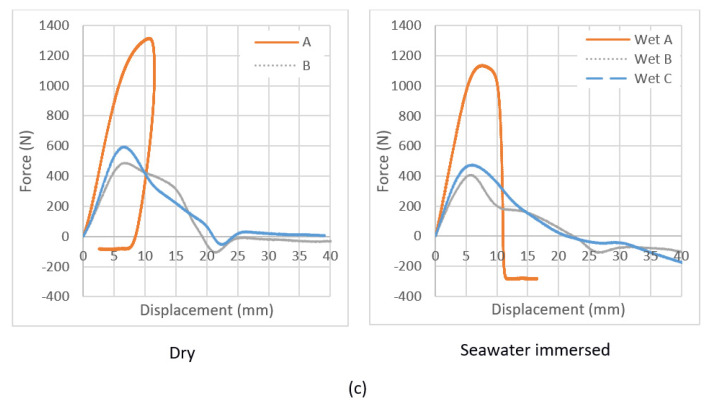
Impact testing results (**a**) energy–time traces, (**b**) force–time traces and (**c**) force–displacement traces at dry and seawater immersed, room temperature conditions.

The impact test was performed on square specimens with dimensions of 100 × 100 mm at an incident impact energy of 10 J for two different environmental conditions, dry and seawater immersed at room temperature. The average values of the impact test results for three different samples with different environmental conditions are presented in the forms of typical energy–time, force–time and force–displacement traces, as shown in Figure 9. Generally, sandwich composites are sensitive to impact loadings. The drop weight impact test results illustrate that the energy absorbed by material A is higher than the other two materials. The peak force from material C is higher than from material B, which indicates the material with paper offers higher resistance during impact. This behaviour could be attributed to the better stiffness of sample C than that of sample B because of the higher thickness of the cork, which contributes to the stiffness. The presence of the paper layers helps to preserve the cork structure. The paper insulates the PLA and helps to maintain the highest cork thickness and better stiffness. During the manufacturing of the laminates, under the effect of the processing pressure, the cork particles move away from each other to accommodate the PLA particles. When the PLA ingresses the cork cavities (without paper), the cork core becomes more fragile and loses its property of impact resistance. 

**Figure 10:**
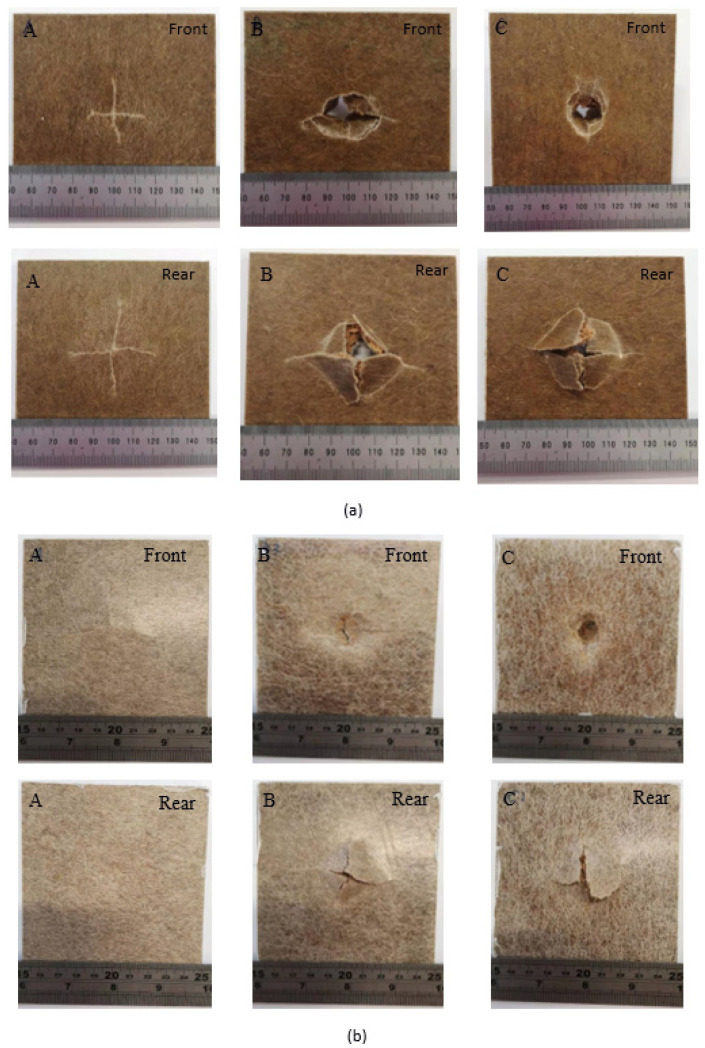
Damaged samples following the drop weight impact testing for three different materials (A, B and C) for (**a**) dry and (**b**) seawater immersed at room temperature.

From the impact images (Figure 10) and from the load–displacement curves, the applied impact energy of 10 Joules was not sufficient to perforate specimen A, force–displacement curves showing closed configuration, an indication of impactor tup rebounding. The localised impact damage can be observed for specimen A. The damaged surface of impacted specimens (samples B and C) shows samples just penetrated, 10 Joules incident energy is sufficient to damage the specimens, which was not the case for sample A. It is plausible to suggest that the flax skins in sample A were capable of withstanding the incident impact energy of 10 Joules, a threshold impact energy. Flax fibre composites are stiff materials, and flax face sheets are accountable for the impact load-bearing, whereas cork core is responsible for energy absorption. Whereas the minimum impact energy required to penetrate the samples B and C is about 6 Joules, approximately a 40% lower minimum energy required compared to sample A. This is related to weak interfacial strength for samples B and C in comparison to sample A. It is worth mentioning here that cork cores also play an important role when considering impact energy absorption for sandwich composites. 

**Figure 11:**
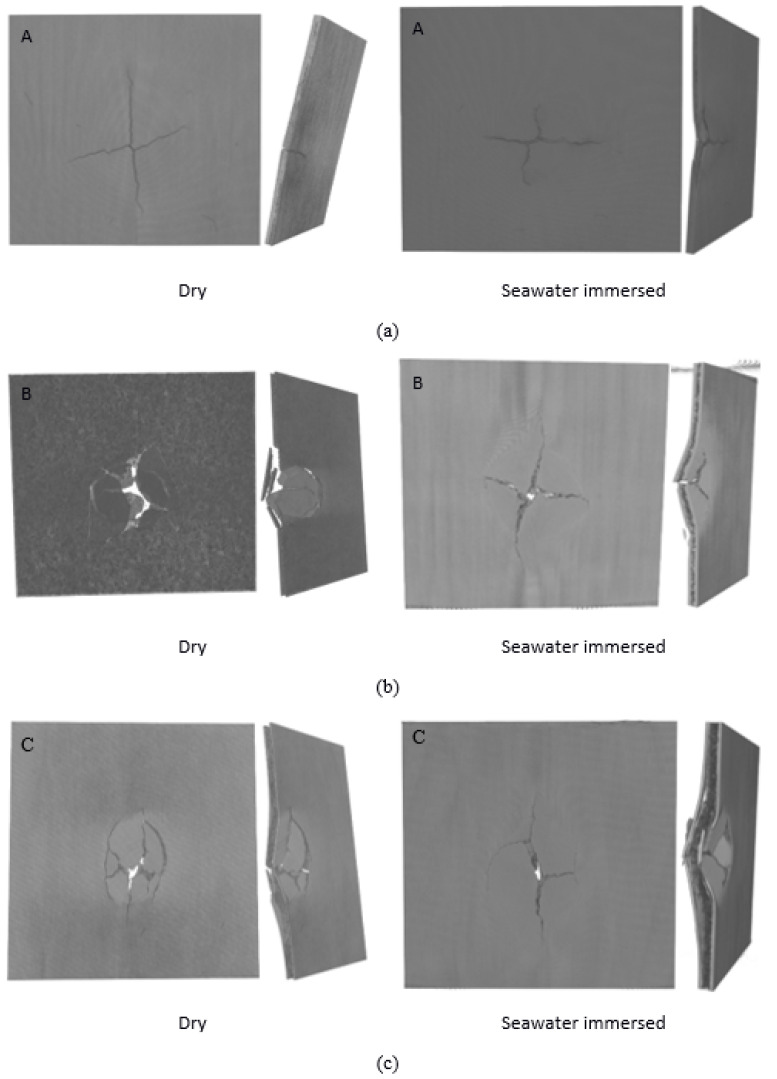
(**a**–**c**) X-ray µCT micrographs of the samples depicting their impacted zones and resultant microscopic cracks in plan and side views for composites A, B and C, respectively, under different conditions dry condition and seawater immersed at room temperature.

The impact damage mechanisms of different samples were first investigated analysing the visible damage seen on the impacted front and rear surfaces of different samples with a naked eye. Figure 10 depicts the front and rear faces of impact damaged specimens that occurred in these composite samples that involve different fracture modes, as shown in Figure 10. Apart from sample A, both samples B and C have been perforated. For sea water-immersed specimens, samples B and C showed significantly less damage. Relating to impact energy absorption, the best performance was displayed by sample A in both dry and wet conditions. Samples B and C showed higher damage in comparison to sample A. It is well established that moisture absorption affects the mechanical properties of composites, as shown by the tensile and flexural test results. However, according to the results obtained, the impact energy is not as severely influenced as seen in the tensile and flexural test results. This is attributed to the higher impact dissipation capability of flax fibres due to their hollow structures and weak fibre matrix interface, suitable for energy dissipation. It is clear that moisture-exposed specimens displaced higher impact toughness than of dry specimens. This observation was confirmed by X-ray µCT micrographs for the impacted samples, as shown in Figure 11. Figure 11 provides the damage on the front faces and the internal cross-sections. 

## 4. Conclusions

The effects of moisture absorption on the mechanical properties and damage mechanisms of a novel flax fibre-based sandwich biocomposites have been presented in this work. The sandwich structure is made of flax composite as the skins and cork as the core. It is evident from the experimental results that the presence of a paper layer in the skins and a cork in the core has a positive influence on the mechanical properties of the sandwich biocomposites. The inference drawn from the observations of the results are summarised as follows:

The moisture absorption for all the materials becomes stable after 1200 h with different environmental conditions. Material A without any paper and cork has the lowest water uptake.

Water-immersed sandwich biocomposites (materials B and C) exhibited inferior tensile and flexural properties for all types of sandwich composites but was more dominant for sample C. This was due to deteriorated interfacial strength due to moisture ingress.

The flexural stress and modules of sample A from this study is comparable to similar studies conducted for flax/PLA in other studies.

The scanning electron microscopy (SEM) images have revealed that the failure modes observed are a combination of matrix cracking, delamination and cork cell breakage.

Based on the above observations, it can be concluded that a cork core with flax/PLA face sheets are promising sustainable biocomposites, which show a significant potential for lightweight applications. The use of cork can be an interesting alternative solution, as it allows the use of laminates with more thickness. The cork is a natural co-product and a cheaper material, produced without any or with a very small energy consumption compared to the PLA. 

## Figures and Tables

**Table 1:** Average tensile test results for three different materials A, B, and C; SD represents the standard deviation of the mean.

Scheme		Tensile Modulus (GPa) (±SD)	Tensile Strength (MPa) (±SD)
A	Dry	3.77 ± 0.13	44.97 ± 0.7
	Wet	2.20 ± 0.3	32.26 ± 1.69
		(a decrease of 41.64%)	(a decrease of 28.26%)
B	Dry	1.61 ± 0.18	26.75 ± 4.45
	Wet	1.42 ± 0.14	21.64 ± 4.13
		(a decrease of 11.8%)	(a decrease of 19.1%)
C	Dry	2.37 ± 0.11	30.29 ± 1.05
	Wet	1.14 ±0.23	17.95 ± 4.20
		(a decrease of 51.9%)	(a decrease of 40.74%)

**Table 2:** Average flexural test results from three different materials A, B and C; SD represents the standard deviation of the mean.

Samples		Flexural Modulus (GPa) (±SD)	Maximum Flexural Stress (MPa) (±SD)
A	Dry	17.74 ± 1.84	162.79 ± 3.12
	Wet	2.30 ± 0.2	64.17 ± 11.86
		(a decrease of 87.03 %)	(a decrease of 60.58 %)
B	Dry	5.46 ± 0.16	48.30 ± 0.98
	Wet	0.70 ± 0.10	17.77 ± 1.29
		(a decrease of 87.18)	(a decrease of 63.21%)
C	Dry	2.34 ± 0.17	30.21 ± 1.58
	Wet	0.43 ± 0.02	7.75 ± 0.32
		(a decrease of 81.62%)	(a decrease of 74.35%)

## Data Availability

Data available upon request to authors.

## Sample Availability

Samples of the compounds are available from the authors.
